# Bacteria-Mediated Oncogenesis and the Underlying Molecular Intricacies: What We Know So Far

**DOI:** 10.3389/fonc.2022.836004

**Published:** 2022-04-04

**Authors:** Shashanka K. Prasad, Smitha Bhat, Dharini Shashank, Akshatha C. R., Sindhu R., Pornchai Rachtanapun, Devananda Devegowda, Prasanna K. Santhekadur, Sarana Rose Sommano

**Affiliations:** ^1^ Department of Biotechnology and Bioinformatics, Faculty of Life Sciences, Jagadguru Sri Shivarathreeshwara (JSS) Academy of Higher Education and Research (JSSAHER), Mysuru, India; ^2^ Department of General Surgery, Adichunchanagiri Institute of Medical Sciences, Mandya, India; ^3^ Department of Medical Oncology, Jawaharlal Institute of Postgraduate Medical Education and Research, Puducherry, India; ^4^ Department of Microbiology, Faculty of Life Sciences, Jagadguru Sri Shivarathreeshwara (JSS) Academy of Higher Education and Research (JSSAHER), Mysuru, India; ^5^ School of Agro-Industry, Faculty of Agro-Industry, Chiang Mai University, Chiang Mai, Thailand; ^6^ Cluster of Agro Bio-Circular-Green Industry (Agro BCG), Chiang Mai University, Chiang Mai, Thailand; ^7^ Centre of Excellence in Molecular Biology and Regenerative Medicine (CEMR), Department of Biochemistry, JSS Medical College, JSS Academy of Higher Education and Research (JSSAHER), Mysuru, India; ^8^ Department of Plant and Soil Sciences, Faculty of Agriculture, Chiang Mai University, Chiang Mai, Thailand

**Keywords:** oncogenesis, chronic inflammation, *Helicobacter pylori*, bacteria, carcinogen

## Abstract

Cancers are known to have multifactorial etiology. Certain bacteria and viruses are proven carcinogens. Lately, there has been in-depth research investigating carcinogenic capabilities of some bacteria. Reports indicate that chronic inflammation and harmful bacterial metabolites to be strong promoters of neoplasticity. *Helicobacter pylori*-induced gastric adenocarcinoma is the best illustration of the chronic inflammation paradigm of oncogenesis. Chronic inflammation, which produces excessive reactive oxygen species (ROS) is hypothesized to cause cancerous cell proliferation. Other possible bacteria-dependent mechanisms and virulence factors have also been suspected of playing a vital role in the bacteria-induced-cancer(s). Numerous attempts have been made to explore and establish the possible relationship between the two. With the growing concerns on anti-microbial resistance and over-dependence of mankind on antibiotics to treat bacterial infections, it must be deemed critical to understand and identify carcinogenic bacteria, to establish their role in causing cancer.

## Introduction

Cancer, one of the leading causes of morbidity and mortality in the world, is characterized by the uncontrolled growth of cells with potential to metastasize. Problems arise when these cancerous cells, carrying mutagenic DNA, turn into tumors (1). The World Health Organization (WHO) estimates that ~10 million deaths occurred due to cancer in 2020 alone (2), twice the number of global COVID-19 related deaths in the same year. Numerous causes of cancer have been identified, with enormous interlink between environmental and genetic factors (3). The alterations occurring in the genetic makeup are known to be influenced by various external factors mostly related to lifestyle, such as alcohol, tobacco abuse, and exposure to sunlight (3). In 2018, roughly 19% and 2% of cancers worldwide had been attributed to tobacco and alcohol intake respectively (4). Interestingly, microbial infections have also been recognized to potentially cause cancer(s) (5–7). According to a 2021 report of the International Agency for Research on Cancer (IARC), there were 2.2 million cancer cases globally related to microbial infection(s), caused by *Helicobacter pylori*, Human papillomavirus (HPV), Hepatitis B virus (HBV), Hepatitis C virus (HCV), and *Schistosoma haematobium* (8).

Traditionally, bacteria have not been considered as a significant etiologic factor for cancer. Though an infectious cause was suspected in the 16^th^ century, the relationship between bacteria and cancer was not very clear due to many reasons. One such example is the varied duration between the onset of infection and the diagnosis of cancer, making it difficult to single out (9). Cancer causing bacteria modulate a variety of immune responses which are believed to play a role in tumor progression. However, the very mechanism of carcinogenesis by bacteria is yet to be elucidated. Notwithstanding, the bacteria-associated factors that may influence neoplasm are not well understood. Carcinogenesis is also influenced by the duration of infection (acute or chronic infections). While epidemiological evidence suggests a reduced risk of cancer in case of acute infections, persistent infections may increase the risk (10). The neoplastic potential of bacterial infections is reported to be influenced by various factors, such as the host immune response, presence of the bacterial toxin, etc. (11). A few bacterial infections are known to promote inflammatory responses amounting to mutagenesis (12), whereas the others are observed to impede the host cell signaling pathways (13). In addition, bacteria interact with host cell(s) and modulate their cell adhesion and cytoskeletal functions (13). This complex network in which a bacteria can possibly promote oncogenesis includes modified cell proliferation and death, alteration of the immune response, and change in the host metabolic processes (14). Recent findings have confirmed the vitality of inflammation in tumor growth promotion, with a direct causal relationship between the two (15, 16). Infection, persistent irritation, and inflammation, in combination, contribute to the development of cancer. In 2011, amongst other cancer hallmarks, tumor-promoting inflammation was highlighted as an enabling trait (17). Furthermore, non-steroidal anti-inflammatory drug use was linked to a lower chance of acquiring various tumors and a lower mortality rate, emphasizing the importance of inflammation in neoplastic transformations (18). Carcinogenesis and inflammation are both highly complicated processes relying independently on multiple signaling mechanisms. Advances in inflammation research have revealed a link between the inflammatory processes and neoplastic transformations, tumor growth, as well as the development of metastases and recurrences (16). The tumor microenvironment, predominantly regulated by inflammatory cells, has now been recognized as an essential participant in the neoplastic process, supporting the proliferation, survival, and migration events. Additionally, innate immune signaling molecules, such as selectins, chemokines, and their receptors, have been co-opted by tumor cells for the purposes of invasion, migration, and metastasis (15).

Surprisingly, the principal global research focus has been limited to establishing the nonspecific mechanisms of carcinogenesis by different microorganisms, including inflammation and toxic bacterial metabolites, rather than understanding the cancer-causing potential of any specific microbe. *Helicobacter pylori*, alongside other bacteria such as *Chlamydia trachomatis*, *Propionibacterium acnes*, and *Fusobacterium nucleatum* have been studied for their associations with cancer (**Table 1.1**). Though many hypotheses have been proposed based on findings from *in vivo* research, the function of persistent inflammation in bacterial oncogenesis has been most widely researched. In addition, specific bacterial virulence factors aiding infection establishment have been examined for their role in oncogenesis. The current review focuses on the various pathways examined in bacterial oncogenesis, taking into account the most widely researched bacterial infection models.

**Table 1.1:** Bacteria implicated in oncogenesis; factors and mechanisms facilitating oncogenesis.

Bacteria	Associated cancer(s)	Reference(s)
*Helicobacter pylori*	Gastric adenocarcinoma MALT Carcinoma	(19) (20, 21)
*Chlamydia trachomatis*	Cervical cancer	(22, 23)
*Neisseria gonorrhoeae*	Prostate cancer	(24, 25)
*Propionibacterium acnes*	Prostate cancer	(26–29)
*Fusobacterium nucleatum*	colorectal cancer	(30, 31)
*Bacteroides fragilis*	colorectal cancer	(32, 33)
*Mycoplasma hominis Mycoplasma genitalium*	Prostate cancer Prostate cancer	(34–37)

## *Helicobacter pylori* (previously *Campylobacter pylori*)

### In Gastric Adenocarcinoma

*Helicobacter pylori* (*H. pylori*) is the first bacterium to be termed carcinogenic by the IARC in 1994 (19). Its infection and relevance with respect to gastric adenocarcinoma are the best studied amongst all cancer causing bacteria. It is an excellent example of cancer caused by bacteria *via* the inflammatory mechanism. It was estimated that nearly one-fifth of all cancers worldwide are due to infections, and *H. pylori* could be implicated in more than 50% of the gastric cancer cases reported (38). The gram-negative bacterium *H. pylori* colonize the stomach and, despite being present on normal stomach epithelial cells, can result in an infection accompanied with inflammation, which, once established, can last for decades (39, 40). This substantiates the role of *H. pylori* as a potent risk factor that can increase the probability of cancer incidence.

An extensive study conducted by the EUROGAST study group found a statistically significant relationship between the incidence of gastric cancer, death rate, and the presence of anti-*H. pylori* antibodies in the serum in 13 countries which is also supported by studies from other researchers (41, 42). In several supporting studies, elevated serum IgG levels were found against *H. pylori* suggesting an infection even without isolation of the causative organism (43, 44). In up to 5% of patients per year, persistent inflammation of the superficial portion of the gastric mucosa is documented to evolve into chronic atrophic gastritis characterized by an advanced cancerous lesion (45, 46). The cancer risk rises 9-fold with substantial atrophy (47). Despite the absence of *H. pylori* in areas with atrophic gastritis, it has often been identified in non-atrophic regions of the same stomach (48).

The probability of malignancy is relatively high with exposure to *H. pylori* infection, as persistent inflammation induces superficial gastritis (19).Oncogenesis promoted by the gram-negative, microaerophilic, spiral bacterium includes several factors comprising cytotoxin-associated gene A (CagA), vacuolating cytotoxin A (VacA), and ROS interactions. Promoters, such as the CagA, VacA, and CagY genes, lead to a higher proliferation of cells or affect gene expression and cell differentiation (49). *H. pylori* is believed to reside in the host for prolonged periods worsening inflammation translating into an increased chance of errors during DNA replication in proportion to cell proliferation, resulting in a cycle of damage, repair, proliferation, and eventually cancer.

Oxidative stress: Greater damage due to oxidative stress is linked to *H. pylori* infection in gastric cells (50). The consequences of oxidative stress upon gastric cells are documented *via* changes observed in the lipid and protein expressions and biomolecular damage (51, 52). Upon infection with *H. pylori*, the epithelial cells of the stomach release ROS, nitric oxide, and chemokines that triggered the production of proinflammatory cytokines, such as interleukin-8 (IL-8), which have been identified as effectors of the inflammatory role in the induction and promotion of the oncogenic process(es) (53, 54). There is also release of interleukin-6 (IL-6), an anti-apoptotic factor, which plays a crucial role in triggering critical signaling pathways, including the activation of JAK, STAT3, PI3K, MAPK, and AMPK ultimately leading to inflammation (55). *H. pylori* induce the Signal Transducer and Activator of Transcription 3 (STAT3) protein activation *via* ROS generation leading to increased expression of the interleukins -6 (IL-6) and -11 (IL-11) (56, 57). The induction of Type 1 T helper (Th-1) cellular response results in the activation of cytokines including gamma interferons (IFN-γ), and interleukin-1 (IL-1) among others, thus resulting in inflammation, loss of healthy host cells, and compensatory cell proliferation (58, 59). With a rising rate of proliferation, errors during replication and accumulation of mutations result from oxygen-free radical accumulation. 8-hydroxy-2’-deoxyguanine (8HdG), an end product of oxidative damage by ROS, leads to transversion of guanine to thiamine in the DNA (59–61). Some reports suggest that the mucosal surface of patients with infection had a higher percentage of 8HdG than those lacking the infection. The levels of this marker are found to be proportional to the infection, as the infection subsides, the 8HdG levels also return to nil, speculating the mutagenic nature of both the bacterium and its metabolite (62). Thence, the inflammation theory of *H. pylori*-induced-oncogenesis may be assumed true. Nonetheless, the other theories, including the activation of the mitogenic transduction pathway, have not been ruled out (63).

CAG PAI: Pathogenicity Island(s) (PAI), a part of the genome carrying virulence genes in pathogenic bacteria, are often absent in non-pathogenic isolates of the same bacteria. The PAI, first described in 1996, was reportedly obtained by the bacteria *via* horizontal transfer, and based on its presence, strains have been categorized into either the very virulent type 1 or the mildly virulent type 2 strains (64). The function of cytotoxin-associated gene pathogenicity island (CAG PAI) of *H. pylori* has been identified as one of the virulence factors in gastric cancer (65). The PAI is responsible for a type 4 secretion system that enables the insertion of CagA protein into the host cells (59, 66). CagA is made of 5 different amino acids, Glu-Pro-Ile-Tyr-Ala, together named EPIYA, occurring either as the EPIYA-D motifs or the multiple EPIYA-C phosphorylation sites, which are associated risk factors for gastric cancer or peptic ulcer disease (PUD) (67).

Once the CagA protein is transferred to the epithelial cells, interaction with host cell proteins, in both phosphorylation-dependent and independent manner, leads to the activation of various signaling pathways involved in cell elongation and scattering, eventually causing responses of the carcinogenic nature (68). Once internalized, CagA can also produce an inflammatory response leading to the release of cytokines such as the IL-8 and -6 *via* activation of the nuclear factor kappa B (NF-κB) (69, 70). Inside the cell, phosphorylation occurs by means of Src and Abl kinases (71), and the phosphorylated CagA activates Src homology-2 domain containing protein tyrosine phosphatase-2 (SHP2), further activating the extracellular signal-regulated kinase (ERK) pathway increasing its activation time with phosphatidylinositol 3-kinase (PI3K), leading to the reorganization of actin, and cellular elongation (72). The phosphorylated CagA interacts with the Src homology 2 (SH2) domains of SHP2, C-terminal Src kinase (CSK), growth factor receptor-bound protein 2 (Grb2), and CT10 regulator of kinase (CRK) proteins (73) containing protein tyrosine phosphatases (PTPs). Thereby causing activation of many tumorigenic signaling cascades by CagA, such as the Ras/Raf/Mitogen-activated protein kinase/ERK kinase (MEK)/extracellular-signal-regulated kinase (RAS/ERK), canonical Wnt pathway (WNT/β-catenin), Janus kinases/signal transducer, and activator of transcription (JAK/STAT), phosphatidylinositol 3-kinase/RAC-alpha serine/threonine-protein kinase (PI3K/AKT), and others along with the inhibition of tumor suppressors such as the tumor protein p53 and ultimately lead to a mitogenic response which is achieved by activation of the PI3K/AKT and ERK/mouse double minute 2 homolog (MDM2) pathways (74–76).

The CagA+ strain infection has been known to cause strong inflammation and damage to the gastric tissues (77, 78). It is noteworthy that these oncogenes were activated only by the positive strains of *H. pylori* (79). The proto-oncogene tyrosine-protein kinase activity is also inhibited by CagA, resulting in the dephosphorylation of tyrosine (80). Similar results were observed due to a defeat in the induction of cell retraction notwithstanding, the signaling molecules responsible have not been identified yet (81). The kinases are phosphorylated in the nucleus, thus triggering the transcription of E-26-like protein-1 (Elk-1) (82), which binds to the serum response factor and subsequently to the serum response elements and stimulates the oncogenic c-Fos and c-Jun upregulation (83, 84). Together, these genes express the activator protein-1 (AP-1) transcription factor, thereby promoting the expression of other late genes responsible for cell proliferation (85). The AP-1 transcription factor activates the transcription of cyclin D (86). In turn, augmented cyclin D activity results in the ultimate release of E2F transcription factors, which *via* cyclin E upregulation prompts the entry into the S-phase (87, 88).

CagA was found to trigger anti-apoptotic responses due to interaction with the p53 protein and thereby causing mutagenesis (89). A significant number of factors and pathways, including the kinases Akt and ERK, anti-apoptotic factors of the B-cell lymphoma family, including MCL-1, BCL-2, and BCL-Xl, were reportedly modulated by CagA (90–93). Furthermore, other proapoptotic factors are majorly involved in the downregulation of autophagy and increase of inflammation, such as Bcl-2-like protein 11 (BIM), BCL2 associated agonist of cell death (BAD), and the apoptosis regulatory SIVA1 are suppressed (91). Recently, the Siva1 protein was identified as a possible factor downregulated by CagA *via* the PI3K/Akt pathway to cause apoptosis inhibition alongside DNA damage (94). the apoptosis-stimulating protein of p53 2 (ASPP2), a critical CagA target and another tumor suppressor found in humans, aids in the survival of the CagA-positive *H. pylori* in the lumen. Notwithstanding, the molecular basis mediating disruption of gastric epithelial cell-polarity observed in the above event and subsequent oncogenesis is yet to be fully understood (95). Various mechanisms of CagA mediated gastric carcinogenesis have been summarized in **Table 1.2**.

**Table 1.2:** Mechanisms of CagA-mediated gastric carcinogenesis.

Factors influenced by CagA	Mechanism	Outcomes	References(s)
Apoptosis-stimulating protein p53 2	Damage to mucosal barrier	Survival of bacteria	(95)
Heat shock protein 1 (HSP1)	Downregulation of HSP1	Persistent infection	(96)
Reg3	Alters the cell cycle	Gastric carcinogenesis	(97)
Caudal type homeobox 1 (CDX1)	Expression of CDX1, promotes the cell proliferation	Gastric carcinogenesis	(98)
Siva1 protein	Activation of the PI3K/Akt pathway	Inhibition of apoptosis, Survival of damaged cells	(94)

**Figure 1:**
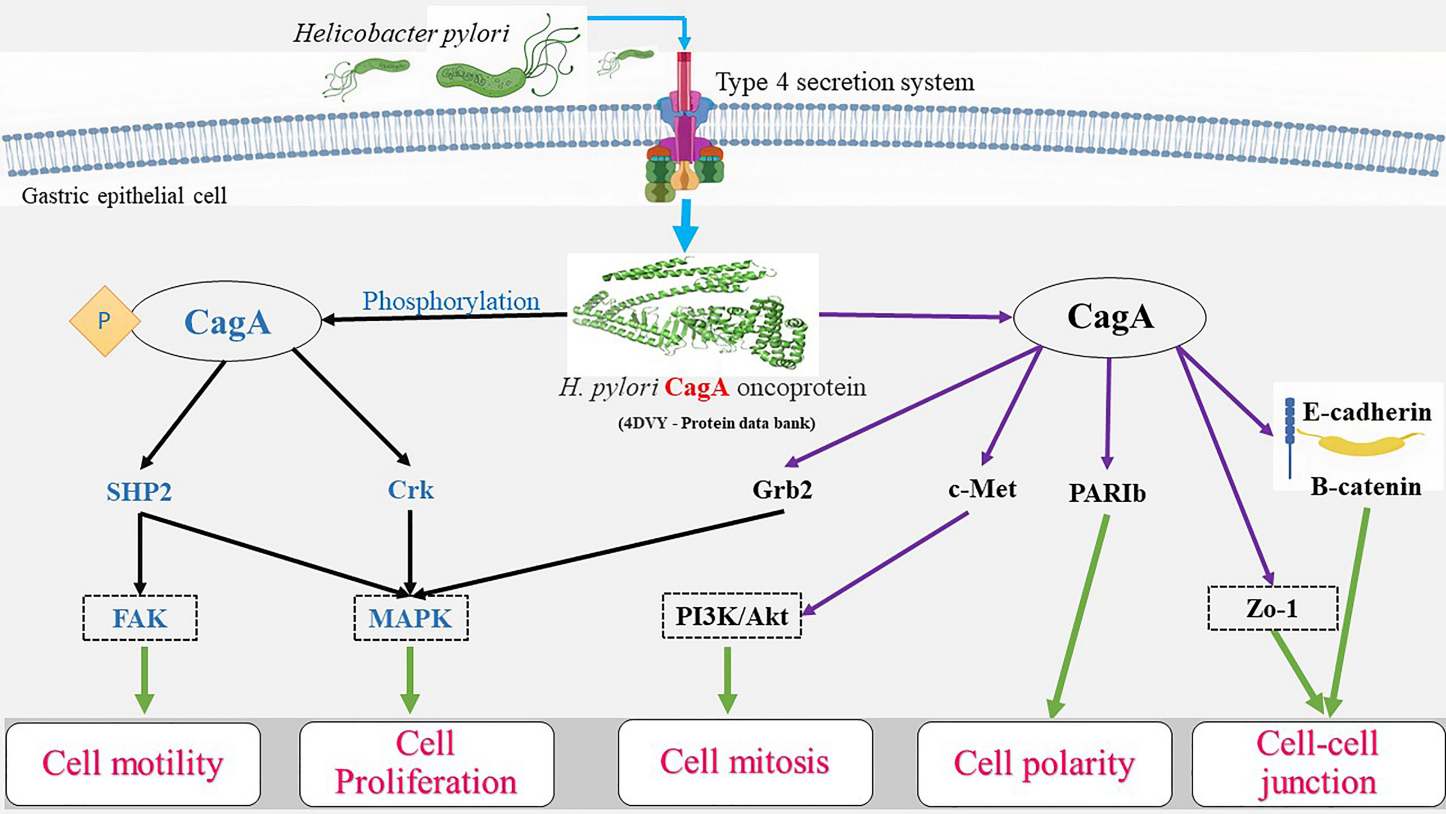
Roles of phosphorylated and non-phosphorylated cagA in neoplastic transformation.

In addition, a few other studies have observed the effects of non-phosphorylated CagA in host cells contributing to pathogenesis. Once inside the gastric cells, non-phosphorylated CagA interacts with E-cadherin leading to the disassociation of E-cadherin and β-catenin complex, amounting to the latter accumulation cytoplasm and nucleus (99). Zonula occludens-1 (ZO-1) and Junctional adhesion molecules (JAMs) interact with CagA and E-cadherin, resulting in junctional instability as well as β-catenin activation (100). Disruption of apical-junction complex (AJC) clubbed with a loss of cell polarity is achieved *via* translocation and activation of beta-catenin (100). It is known to target E-cadherin, tyrosine-protein kinase Met (c-Met), and kinase partitioning defective 1b (PAR1b) or microtubule affinity-regulating kinase 2 (MARK2), resulting in inflammation and mitogenesis (100, 101). The β-catenin and T-cell factor complexes formed trigger the expression of genes that encode cyclin D1 and cellular myelocytomatosis oncogene (c-Myc), leading to abnormal cell proliferation (102). Non-phosphorylated CagA also brings about alternations in cell motility and proliferation by binding to GRB/SOS/RAS and activation of Raf/MEK/Erk pathway, joining with ZO-1 and JAM-A tight junction proteins. The effects of phosphorylated as well as non-phosphorylated CagA in gastric neoplasm has been illustrated in **Figure 1**. 

The number of CagA-positive *H. pylori* strains varies greatly among geographic regions. While almost all variants can be found in the East Asia, there are less than half prevalent in the west (103). The CagA-positive strains of *H. pylori* have been classified as the East Asian and the Western types based on the 3’ end region made of repeating sequences containing EPIYA phosphorylation site. Where, the former constituted EPIYA-A and EPIYA-B segments, and the latter contained EPIYA-C and EPIYA-D, respectively (104). EPIYA-D type segments were found to have more remarkable *in vitro* SHP-2 binding ability (104). In the transgenic mice model, the carcinogenic potential of CagA has been questioned concerning the positive and negative species of *H. pylori*, highlighting CagA as a potential oncoprotein (105). It is widely accepted that CagA-positive *H. pylori* are related to a greater risk of gastric cancer, however, the same outcome has not been seen in CagA-negative *H. pylori* (106). Notwithstanding, irrespective of the strain used, researchers failed to induce gastric cancer in the Mongolian gerbil model (107, 108).

VacA: Vacuolating cytotoxin (VacA) and those proteins linked with the outer membrane of *H. pylori* are involved in the process of vacuolation and ulcer formation (109). VacA, secreted by the type 5 secretion system in all isolates of *H. pylori*, is present in the mitochondria and affects its functions (110, 111). Initially formed as a 140kDa precursor, it matures to become an 88 kDa protein comprising p33 and p55 (112). The p55 domain is mainly responsible for the building of cell surface receptor proteins such as the tyrosine phosphatase (RPTP), epidermal growth factor (EGF), sphingomyelin, and fibronectin (113), while the p33 domain forms a channel of 6 subunits of VacA to facilitate chloride transport. This protein can separate the tight junction of gastric epithelial cells, thereby crossing the barrier (114). Once bound to the cell, VacA enters it by a mechanism independent of clathrin (115, 116). Many cell-surface components such as the RPTP-α (117), RPTP-β (118), various lipids (117), heparin sulphate (119), sphingomyelin (120), as well as Integrin beta chain-2 (integrin β2; CD18) on T cells (120) are targeted by VacA. Notwithstanding, the roles played by these factors in VacA uptake remain unidentified. Vacuolation of cells, disruption of apoptosis and lysosomal functions are some of the most important alterations caused by VacA cytotoxicity (121). Vacuole formation is achieved by means of *in vitro* endosomal compartment(s) disruption (122).

**Figure 2:**
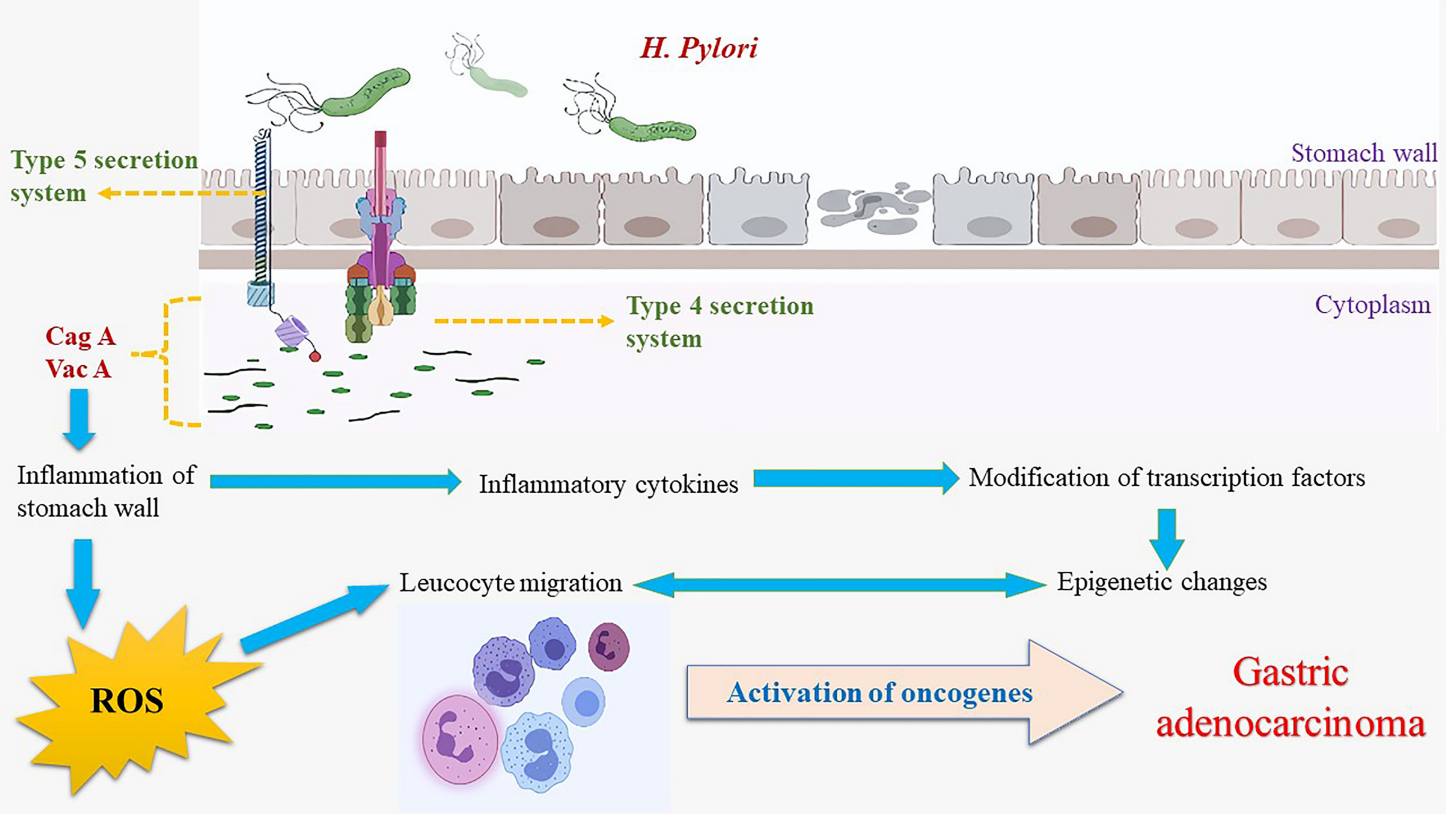
Etiopathogenesis of gastric adenocarcinoma with *H. pylori* infection.

VacA activates akt *via* phosphatidylinositol 3-kinase dependent phosphorylation of glycogen synthase kinase – 3 beta (GSK3β) (123). Akt phosphorylation and activation are achieved *via* two protein kinases 3-Phosphoinositide-dependent kinase - 1 (PDK-1) and mammalian target of rapamycin complex 2 (mTORC2) (124). In VacA affected cells, inhibition of Rapamycin complex 1 (mTORC1) signaling positively regulates autophagy as well as affects the host cell metabolism and stress signaling (125). Cell death occurs *via* the Unc-51like autophagy activating kinase – 1 (ULK1) complex, using the low-density lipoprotein (LDL) receptors (125). Hence, Akt phosphorylation inhibits GSK3β and subsequent proliferation and survival (126, 127). GSK3β phosphorylates β-catenin in a cytoplasmic complex constituting auxin, adenomatous polyposis coli (APC) protein, and β-catenin in the absence of the ligand (128). The phosphorylated β-catenin is then ubiquitinated and destroyed by the proteasome (129). GSK3β remains inactivated in the presence of VacA, causing β-catenin accumulation in the cytoplasm (130). The β-catenin protein serves as a transcription factor coactivator, T cell factor, and lymphoid enhancer factor upon entering the nucleus to activate transcription of the β-catenin-dependent genes such as the cyclin D1 gene, CCND1, whose overexpression is linked to cancer (102). β-catenin signaling pathway is affected by VacA, presumably having an oncogenic role (131). The association of VacA and CagA in anti-apoptotic signaling may be one of the highly effective strategies of the bacterium to protect itself from the gastric niche and the human immune defense (112). *In vivo* studies involving Mongolian gerbil, models have observed apoptotic loss of pit cells by *H. pylori* and decreased apoptosis leading to hyperplasia and colonization mediated by CagA *via* MAP kinase protein (132). *H. pylori* can cause genomic instability in the gastric cells through epigenetic pathways (133). Previous *in vitro* studies have documented the induction of breakage in DNA strands by irrespective of CagA Presence in strains (134). Other studies have found that CAG PAI resultant products may have a crucial role in the accumulation of DNA strand breaks in the infected gastric cells (135). It was also hypothesized that host-bacterium interaction was responsible for DNA double-strand breaks, postulating that treatment and elimination of *H. pylori* may show reduced gastric cancer risk (136). The overall effect of virulence factors and inflammation on gastric epithelial cells is summarized in **Figure 2**.

### In Gastric Mucosa-Associated Lymphoid Tissue (MALT) Carcinoma

The only human malignancies in which the etiological function of a specific bacterial infection has been broadly established are gastric adenocarcinoma and MALT lymphoma. As many as half of all MALT lymphoma cases are reportedly occurring in the stomach, and *H. pylori* were found to be prevalent in 90% of gastric MALT lymphoma tissues (20, 21). Given the morphological similarities between the follicles amongst gastric MALT lymphoma tissue(s) and those affected by *H. pylori*, a high incidence and direct relation were suspected between *H. pylori* and MALT lymphoma (21). A connection can thus be established between *H. pylori* and gastric MALT lymphoma (137).

As a result of repeated stimulation with *H. pylori* antigens, chronic infections lead to the formation of MALT in the stomach mucosa as they stimulate specific T-cells, marking the early stages of oncogenesis (138, 139). The assistance of tumor-infiltrating T-cells is essential for the development of MALT lymphoma *in vitro* (90). Tumor-infiltrating T-cells promote the proliferation of B cells when stimulated by *H. pylori* (90). Cytokine and CD-40 mediated cell signaling have been observed mandatory for lymphoma formation (90). In MALT lymphoma cells, the B-cell attracting chemokine 1 (BCA-1) and its receptor C-X-C motif chemokine receptor 5 (CXCR5) are augmented, which regulate B-cells and promote the production of the inflammation-causing interleukin-8 (IL-8) (140).

Translocated by the type 4 secretion system, CagA along with the SHP-2 stimulates B-cells *via* p38 kinase (105), bringing about B-cell proliferation *via* the control of endoplasmic reticulum kinases 1 and 2 (ERK 1 and 2) (105, 141). Due to phosphorylation and lowering of SHP-2, CagA promotes *H. pylori*-associated gastric neoplasm formation (142) in murine models. In addition, apoptosis of B-cells can be blocked due to the accumulation of p43 in the presence of CagA (141, 143). Alternations in the p53 suppressor gene influence the grade of lymphoma formed (144). Interference with antigen presentation of B-cells is brought about by VacA, affecting cell proliferation (145, 146). Molecular studies have shown changes in methylation of DNA at cysteine and guanine nucleotides which can subdue the tumor suppressor genes. Another contributing factor is the CpG island methylator phenotype found in 60% of MALT lymphomas due to *H. pylori* infection (147). Notwithstanding, growing chromosomal aberrations may enable MALT lymphomas to exist without an *H. pylori* infection (148).

Epidemiological studies reveal that the host factors such as the amount of salt intake also surged the degree of infection and frequency of cancer (149, 150). Experimental studies suggest synergistic effects of salt on lesions (151), wherein increased salt consumption leads to an augmented expression of CagA (152). These findings shed light on how *H. pylori* avoid inducing excessive cellular damage while maintaining long-term colonization. As observed, activation of cell proliferating signaling pathways was initiated by CagA and VacA. Nonetheless, further studies may be required to study and understand the effects of the inactivation of the above pathways in designing new therapeutic targets.

### Treatment or Elimination of *H. pylori*

Antibiotics and proton pump inhibitors (PPIs) are commonly used in the event of *H. pylori* infection. Clarithromycin triple therapy with clarithromycin and amoxicillin, bismuth quadruple therapy with bismuth and tetracycline, and concomitant therapy with clarithromycin and amoxicillin in combination with a PPI and metronidazole constitute the recommended antibiotic regimens (153).

Meta-analyses of trials have resulted in reduced incidences of gastric cancer with the eradication of the bacteria (154, 155). In another similar trial, follow-up led to the reduced incidence of cancerous lesions after the eradication (156). Yet another trial comprising follow-up after eight years observed a 50% reversal of atrophic gastritis in the bacteria-eradicated patients (157). notwithstanding, the examination of available data indicates that no trials or studies have demonstrated a significant rise or decline in the incidence of cancer post-eradication after the infection is past the atrophic gastritis stage. However, eradication could undoubtedly prevent the development of precancerous lesions (158). This may be indicative of one clinical benefit that eradication at earlier stages of infection could be helpful. The prevailing notion is that eradicating infection before the dysplasia stage could be of benefit (159). Most meta-analyses have noted neither enough evidence nor data to claim any association between *H. pylori* and MALT carcinoma (155, 160). Similar observations have been made in individual studies, where MALT lymphomas were unresponsive to *H. pylori* eradication therapy (161). Once *H. pylori* infection was removed, 83% of the lymphomas were seen to be regressed (137). However, other studies have reported that the eradication therapy could be effective in long-term outcomes for *H. pylori*-induced-MALT lymphomas regardless of the infection stage (162, 163). Indicating the need for a more solid substantiation to link the bacteria’s eradication and cancer regression (164).

## Chlamydia trachomatis

*Chlamydia trachomatis*, an intracellular, obligate, Gram-negative bacterium, is known to cause Chlamydia. While many discovered serovars of this species are known to infect different organ systems, the Serovars A-C instigate infection in the eyes, and serovars D through H colonize the genital tract. As of 2018, Cervical cancer is responsible for nearly 8% of cancer-related mortality, ranking 4^th^ for both incidence and mortality (165). Many *in vitro* and *in vivo* studies reported an association between cervical neoplasm and chlamydial infection (22, 23). Implicated in a heightened risk of uncontrolled cervical cell growth, the presence of the chlamydial infection has also been associated with increased cancer incidence (166–169). Notwithstanding, the association remains controversial as various other reports indicated no alliance between the infection and the development of cancer (170, 171).

While the process of oncogenesis is yet unclear, it is hypothesized to arise from persistent inflammation and metaplasia (172), particularly through the squamous cell metaplasia. *C. trachomatis* has been known to cause cancer which may develop over years or decades (172, 173). As an intracellular pathogen, these bacteria can only multiply inside a host cell by dodging the immune system *via* prevention of phagolysosome formation (174), thereby affecting major histocompatibility complex (MHC) induced antigen expression (175) and its anti-apoptotic properties (176). *C. trachomatis* infection has been documented to modify the transcription of genes responsible for cell differentiation, cell death, and transcription factor(s) expression (177). Chronic inflammatory response(s), modified metabolite production, the amplified activity of cytokines, and decreased cell-mediated immunity contribute to mutagenesis by facilitating uncontrolled, multipolar mitosis and injury to DNA repair systems amounting to accumulation of aberrant DNA and thereby cancer (178, 179). *In vitro* studies evaluating the effects of *C. trachomatis* on apoptosis, inhibition observed unaffected DNA synthesis in the infected cells, which could undergo regular mitosis at any point of infection, linking it to a heightened risk of malignancy (180).

The apoptosis inhibition caused by *C. trachomatis* infection reasons the occurrence of neoplasm (181). Another mechanism through which apoptosis inhibition occurs is *via* mitochondrial cytochrome C inhibition (182). However, three different pathways of achieving this have been theorized. Firstly, by the inhibition of upstream activities controlling mitochondrial function *via* production of anti-apoptotic factors (183, 184). Secondly, Bcl-2 or Bcl-2-like molecule expression may prevent the activation of caspase and cytochrome c production (181) although, the expression of Bcl-2 does not guarantee blockage of apoptosis (185). Lastly, pertaining speculations indicate the involvement of other anti-apoptotic factors which are yet to be identified or understood (181).

Tyrosine phosphorylation of host cell proteins involved in signal transduction pathways is upregulated during *C. trachomatis* infection (177, 186–188). In addition, carcinogenic components of the Ras-Raf-MEK-ERK pathway are observed to be activated by the bacterium along with the ROS production for survival (189–191). The p62 knockdown was found not to affect host cells or autophagy during early infection, notwithstanding, in the later stages of infection, autophagy was affected by p61 silencing as seen *in vitro* (192). Thereby, it may be deduced that p62 has a significant role in bacterium-induced autophagy, providing the necessary supportive data and theoretical basis for further study into bacterial pathogenesis. The plasmid-encoded protein Pgp3 inhibits apoptosis with PI3K/AKT signaling pathway activation, MDM2 (murine double minute 2) phosphorylation, and nuclear entry, as well as p53 degradation (193). In HeLa cells, Pgp3-induced inhibition of apoptosis was hindered, suggesting that the PI3K/AKT pathway had a critical role MDM2-p53 axis in Pgp3 anti-apoptotic activity. Nonetheless, the precise molecular targets and pathways are due to be further identified (193).

The pORF5 plasmid protein plays a crucial role in mitochondrial autophagy and apoptosis by upregulation of knockdown high mobility group box 1 (HMGB1) which may be necessary to *C. trachomatis* in modulating mitophagy whose specific upstream and downstream signaling pathways remain unknown, hence establishing growth (194). Further, 3-phosphoinositide-dependent protein kinase one signaling is evoked by the infection leading to the stabilization and phosphorylation of MYC (195). MYC- PDPK1 signaling activates the hexokinase of host II (HKII), which is moved into the mitochondria. It was found that the prevention of HKII interaction with mitochondria with the use of exogenous peptides triggered the apoptosis of infected cells in a manner similar to inhibition of either PDPK1 or MYC, resulting in disruption of intracellular development of the bacteria (195). The target of the MYC-PDPK1-HKII-axis could be considered a novel scheme in overcoming therapeutic resistance to the infection (195).

Centrosomes and centrosome segregation defects were produced in excess during *C. trachomatis* infection as a result of multipolar cell division, promoting genetic instability (178). Various *in vitro* studies have documented that chlamydial infection led to incremented multinucleation of host cells, directly linked to neoplastic transformation (196). Defects in the mitotic spindle pole were due to heightened supernumerary centrosomes, amounting to apoptosis activation resistance in the cell division cycle and subsequently leading to oncogenesis (178, 180, 181, 197). Furthermore, centrosome amplification and segregation defects in the chromosomes were suggested to promote instability (178). Trigger of supernumerary centrosome production and chromosome segregation defects, multipolar mitosis, chromosome instability promotion, and multinucleation lead to the malignant transformation and subsequent tumor development (178, 180, 198).

### Chlamydial Heat Shock Protein

Heat shock protein-60 (HSP60), a protein-folding protein, is found in the cytoplasm of cells (199). The HSP-60, similar to GroEL of *Escherichia coli*, can induce inflammation. It was proposed that the *C. trachomatis* HSP60 may serve as a risk factor for oncogenesis by the mediation of apoptosis (199). The host cells affected by the chlamydial HSP60 are highly susceptible to oncogene expression for survival, continued proliferation, and eventually malignancy (199). Contradicting this theory, Capello, 1990 (200) proposed the presence of anti-chlamydial HSP60 antibodies providing immunity against cancer. Reports indicate that copious amounts of HSP60 are produced by *C. trachomatis* during infectious stages (201). Some tumors were found to present HSP60 on their surface, bringing about antibodies towards their epitopes in an attempt to induce an anti-tumor response (202). This surplus of chlamydial HSP60 seen in the cytoplasm and the host cell membrane during a long-standing infection promotes activation of immune cells against the protein, followed by endocytosis (203). These endocytosed proteins bind to toll-like receptors (TLRs), resulting in the activation of signaling networks responsible for the proliferation of host cells (204, 205). Protein-mediated anti-apoptotic activity *via* the formation of a complex with Bax and Bak proteins to cut the outer membrane of mitochondria has been documented (206).

A higher incidence of cervical cancer was directly linked to the increased anti-chlamydial heat shock protein antibodies (202). Some studies concluded that the HSP, with anti-apoptotic properties, was blamed for chronic inflammation (207–209). Airenne 2002 and Carratelli 2000 (23, 210) observed that the heat-labile component *C. pneumoniae* is released during infection validates HSP60 as a risk for cancer. The risk of infection increases with time, right from the moment of serum sampling to cancer diagnosis, similar to the serological studies of *H. pylori* in gastric cancer (209). It has also been noted that the different serotypes of *C. trachomatis* show variable risks, and the serotypes B, D, E, G, I, and J have been linked to an increased risk of squamous cell cancer (211).

Emphasizing the need to consider and identify possible cofactors responsible for enhancing cervical carcinogenesis (212). While HPV infections are prominently linked to cervical cancer, reports suggest that only a fraction of these infections are responsible for oncogenesis (213). Much evidence also stipulates that the risk of HPV acquisition and persistence is raised with *C. trachomatis* infection (214). *C. trachomatis* infection history and HPV have been linked in two recent studies, thus confirming the hypothesis of *C. trachomatis* being a cofactor (214). Furthermore, *C. trachomatis* may have a suggested role in aiding HPV in the carcinogenesis *via* MMP-9/RECK imbalance during cervical inflammation as a part of the infection (215).

**Figure 3:**
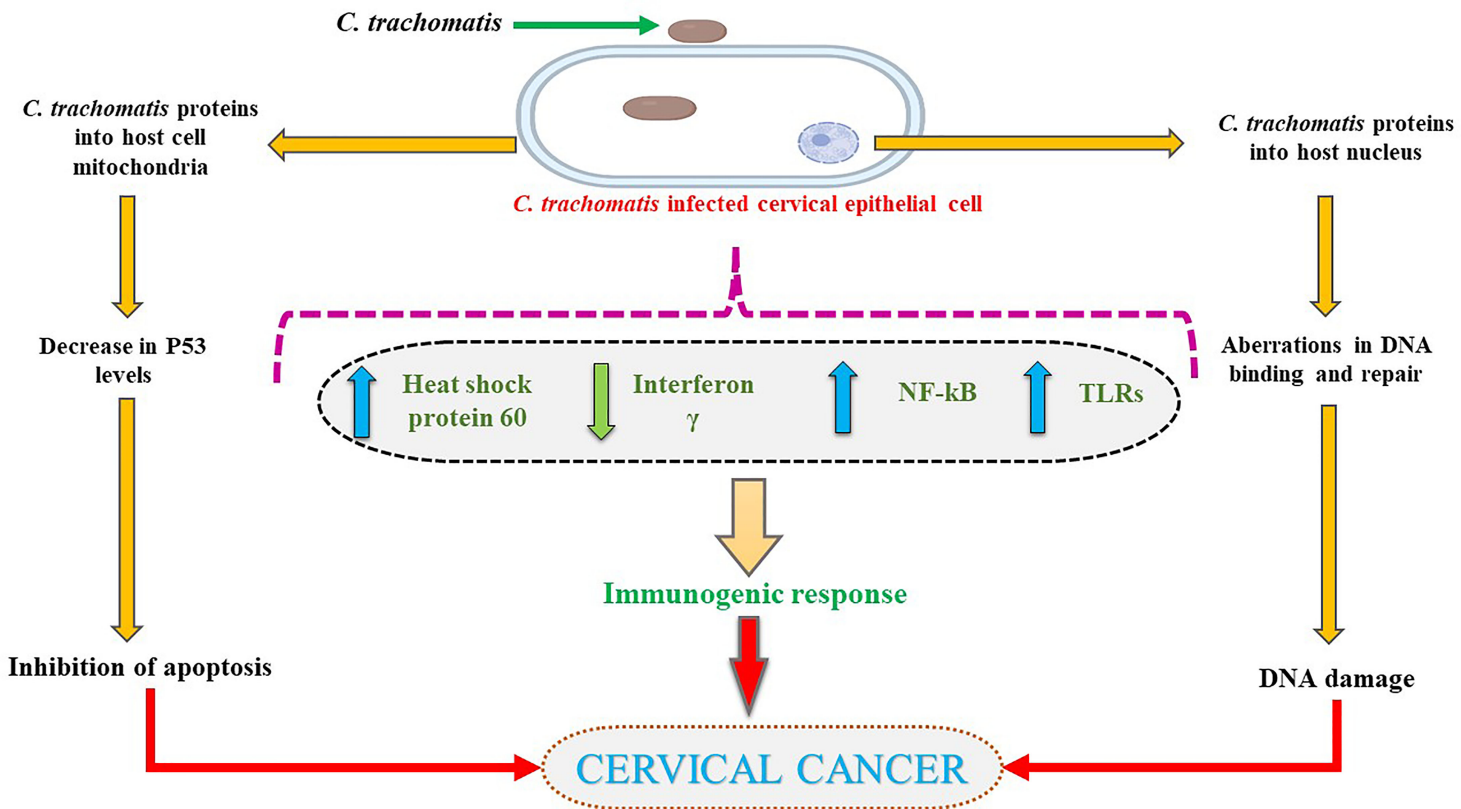
*C. trachomatis* affecting host cervical cells.

Recent meta-analyses have evaluated the use of azithromycin *vs.* doxycycline and found doxycycline to be more effective in treating *C. trachomatis* infection (216). Notwithstanding, the Centre for Disease Control (CDC) recommended treatment regimen for chlamydial infection includes doxycycline, azithromycin, or levofloxacin (217). Therefore, finding more screening techniques and treatment options is deemed necessary for those affected with *C. trachomatis*. The effects of *C. trachomatis* on cervical cells are summarized in **Figure 3**.

### Fusobacterium nucleatum

*Fusobacterium nucleatum* is a gram-negative, non-sporing bacterium capable of forming biofilms, commonly known for causing teeth infection (218). Speculations prevail that it brings about inflammation and invasive infections *via* hematogenous dissemination from the oral cavity to the colon (219–221). Many researchers have hypothesized an association between F. nucleatum and colorectal carcinogenesis (CRC) (30, 31) and considered the bacterium a risk factor for cancer progression. Recent meta-analyses and independent studies have found significantly raised levels of *F. nucleatum* during CRC incidents (222–231). Several other studies have also speculated that *F. nucleatum* may synergistically promote CRC with other bacteria such as the *Streptococcus* spp. and *Campylobacter* spp (232, 233). In an International ColoCare Study, it was observed in non-treated patients that varying levels of the bacterium were found at the tumor sites, indicating its use as a possible prognostic and diagnostic marker in the management of CRC (234).

The bacterium also aids neoplastic transformation *via* obstruction of anti-tumorigenic immunity by recruiting lymphocytes that infiltrate the tumor as well as activating immune checkpoints such as the T-cell immunoreceptor with immunoglobulin and ITIM domains (TIGIT) and Carcinoembryonic antigen-related cell adhesion molecule 1 (CEACAM1), which aid in the inhibition of apoptosis (235–238). Generation of optimum microenvironment and activation of β-catenin signaling are some mechanisms by which *F. nucleatum* is involved in cancer progression (239–241). Recruitment of pro-inflammatory immune cells occurs due to the ROS-rich microenvironment (242, 243). Inflammation is worsened by the NKp46 receptor of natural killer cells in the presence of *F. nucleatum*, which prompts the release of TNF-α (244). Generation of the proinflammatory neoplastic microenvironment, higher rate of cell proliferation *via* Wnt/β-catenin signal activation, and signaling of NF-kB by TLR4 (31, 227, 240, 245) are some of the alternate mechanisms proposed for oncogenesis caused by F. nucleatum. Several *in vitro* studies have associated a higher prevalence of the bacteria with activation of oncogenic molecular cascades, including the instability of microsatellites, genetic mutations of BRAF, CHD7, CHD8, and TP53 CpG island methylator phenotype (246, 247).

The lipopolysaccharides, FadA, and Fap23 molecules present on the bacterium’s surface have been found to instigate oncogenesis (231, 248). Stimulation of malignant cell growth was found occurring due to β-catenin signal induction and tumorigenic gene expression *via* the virulence factor FadA (31, 239). The RadD adhesin, an arginine-instable adhesin, was reported to aid in the bacterial attachment and invasion into host cells, apart from aiding biofilm formation (31, 249). FadA, a virulence protein, monitors the bacterial entrance into host cells by activating the inflammatory and carcinogenic signals to induce growth in cells (250, 251). 2 forms of FadA, the pre-FadA and mFadA have been identified, which as the pre-FadA-mFadA complex are essential for the function as mentioned above (250, 252). FadA affects E-cadherin and β-cadherin, stimulating the T-cell factors and ultimately resulting in the expression of oncogenes, inflammation, and proliferation (31, 253).

Loss of E-cadherin alteration of the Wnt signaling pathway is an essential process in mesenchymal transition (254). In the Wnt pathway, β-catenin is responsible for the downregulation of E-cadherin, leading to the mesenchymal transition (255). Usually existing as a complex at the epithelial surface, β-catenin and E-cadherin are separated and migrated to the nucleus (227), which results in the alternation and deregulation of the Wnt signaling pathway, leading to tumor formation. Furthermore, the levels of the FadA gene in colorectal tissues of infected patients have been observed to be elevated and associated with inflammatory genes (256), hence substantiating the claims that virulence factors of *F. nucleatum* have a possible carcinogenic effect. In an *in vivo* study, the surface galactose-binding lectin, Fap2, was found to mediate *F. nucleatum* recruitment to the CRC cells (251). Furthermore, a polysaccharide D-galactose- β-N-acetyl-D-galactosamine (Gal-GalNAc) has been found in CRC tissues, which bind to FAP2, leading to the enrichment of the bacterium (249). A new miRNA-mediated pathway has been hypothesized by which *F. nucleatum* can affect the host cells and cancer (257). Researchers observed the enlarged tumor rate and decreased survival rates when APC/- mice were fed with *F. nucleatum*. In addition, the co-culture of *F. nucleatum* in the CRC cell lines led to increased cell proliferation *in vitro* and *in vivo* (257). With the theory of miRNA deregulation in colorectal cancer, many researchers studied miRNA expression in the exposed cell lines (245, 258). The engagement of fusobacterial lipopolysaccharide by toll-like receptors led to the induction of miR21, leading to the activation of RAS-MAPK signaling through the miR21 target RasGTPase enzyme (245, 258).

**Figure 4:**
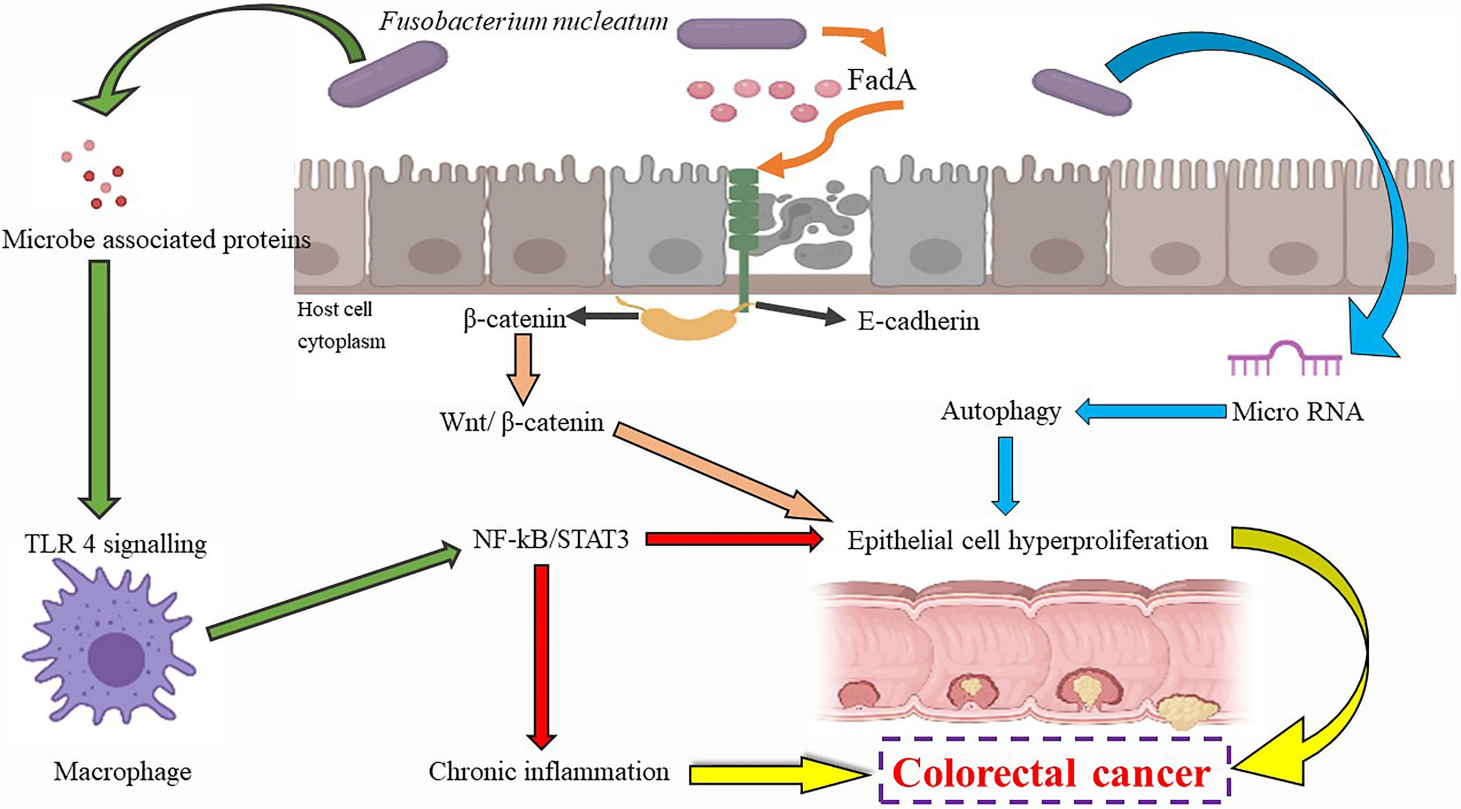
*Fusobacterium nucleatum* and cancer associations.

The putative mechanism of F. nucleatum and its effects are summarized in **Figure 4**. Markers of inflammation such as IL-8 and IL-6 TNF-α have been elevated in case of infection (259). The adhesion of cells, autophagic flux, and anti-tumor activities of immune cells are affected by F. nucleatum, apart from decreasing the activity of T cells in adaptive immunity by affecting the G1 phase (260). While F. nucleatum is noted to promote cell proliferation by modulating E-cadherin and β-catenin pathways, increasing miRNA-21 expression. On the contrary, in the human gingival fibroblasts, F. nucleatum prevents cell proliferation and induces cell death by activating the AKT and NF-kB signaling pathways (260). This bacterium has also been found to reduce chemotherapeutic effects in CRC due to activation of TLR4/NF-κB pathways (261, 262). All these findings together suggest an important role of *F. nucleatum* in cancer initiation. The presence of β-lactamase in a few strains may make these organisms resistant to penicillin, indicating that the anaerobic antibiotics such as metronidazole or clindamycin may be the drug of choice in the treatment of this infection (263, 264).

### Bacteroides fragilis

*Bacteroides fragilis* are non-spore-forming, Gram-negative, anaerobic bacteria constituting two different classes, *i.e.*, non-toxigenic *B. fragilis* (NTBF) and enterotoxigenic *B. fragilis* (ETBF), based on their ability to produce biofilm and the presence of the gene for zinc-dependent metalloprotease, *B. fragilis* toxin (BFT) (32, 265, 266). The infliction of tight junctions and increase in intestinal permeability caused by BFT may be necessary for inflammation of the intestine and, further, in neoplastic transformation (32, 33). *In vitro* tests on HT29/C1 cells with BFT treatment revealed a decline in membrane-associated E-cadherin initiated the nuclear localization of ß-catenin, which further induced translation of c-myc and continuous cell proliferation (267). This ability of BFT to affect the epithelial cells has led to many researchers concluding that the ETBF may contribute towards CRC (266–268). Long-term colonization by ETBF in the intestine results in chronic inflammation stimulation due to activation of STAT3, which leads to increased IL-17 production responsible for prolonged inflammation in the intestine (269). BFT modulates signaling pathways and is responsible for ROS production, leading to mutagenesis and cleavage of E-cadherin (266, 270). It can activate β-catenin signaling and induce IL-8 production in epithelial cells (268). Additionally, being biofilm producers, ETBF degrades E-cadherin in cells, causes the production of IL-6, and activates STAT3 pathways, enhancing cell proliferation. Indicating that biofilms are associated with neoplastic development in the colon (271). While the ETBF through biofilm can induce cancer, the NTBF cannot harm the intestinal tract (272). The ETBF promoting colorectal carcinogenesis, upregulation of JMJD2B, a histone demethylase, *via* TLR4-NFAT5-dependent pathway is caused by the ETBF promoting colorectal carcinogenesis (273).

Further, BFT has been documented to trigger the production of COX-2, which releases prostaglandin E2 (PGE2), which causes inflammation and controls cell proliferation *via* control of signaling pathways. Hence, COX-2 plays a vital role in colon carcinogenesis *via* angiogenesis promotion, stem cell formation, inhibition of apoptosis, increasing metastatic potential, and promotion of cell proliferation (266, 274–278). The serum COX-2 levels have also been used as a biomarker in CRC patients, indicating aggressive growth and higher mortality rates compared to normal individuals (279–281). Downregulation of miR-149-3p by ETBF was found to promote PHF5A-mediated RNA alternative splicing of KAT2A in CRC cells (282).

Via secretion of chemokine IL-17 along with other cell surface receptors, activated through induction of NF-kB pathways, BFT establishes a pro-carcinogenic signaling relay in ETBF-associated carcinogenesis (283). Chemokine motif ligand 3 is a macrophage inflammatory protein with CCR5 as the receptor. CCR5 plays a vital role in invasion and metastasis *via* inflammatory factors and tumor-associated genes to regulate NF-kB (284). Some studies have found that BFT promotes and may be necessary for the proliferation of colorectal cancer due to the acceleration of CCl-3 molecular pathways (285).

Significant associations have been established between the presence of ETBF and colorectal cancer, however, additional research is required to determine other factors affecting their relationship. Due to the presence of β-lactamase, the ETBF is resistant to penicillin. Antibiotics such as cefoxitin and clindamycin have little susceptibility towards the bacterium, while piperacillin/tazobactam, meropenem, and metronidazole are known to be more effective (286).

### Neisseria gonorrhoeae

*Neisseria gonorrhoeae (N. gonorrhoeae)*, the causative organism of gonorrhea, is a gram-negative, facultative intracellular pathogen. A history of infection with *N. gonorrhoeae* has been suggested to be associated with a higher incidence of prostate cancer risk, as reported by a few meta-analyses (24, 25). In 2018, prostate cancer was the second most frequent form of cancer in men worldwide (287, 288). Gonorrheal infection is one of the most common causes of prostate cancer (289).

Although the exact molecular mechanisms in oncogenesis are unclear, chronic and repeated infections of this bacterium have been associated with prostate cancer (290). The duration of infection has been observed to be directly proportionate to a higher risk of cancer (291). Following the infection, a persistent inflammatory phase is induced in the prostate. The bacteria attach to the epithelial cell surface made possible by the type IV pili, the unique appendages on the bacterial surface (292, 293). Once attached, the host cell signaling events occur, eliciting induction of the anti-apoptotic activities (294, 295). A large number of cytokines and chemokines (interleukins 6 and 8) are secreted following the damage due to inflammatory cells that promote oncogenesis (296). Pathological examinations have revealed proliferative atrophy with inflammation which may be a precursor lesion to cancer (296, 297).

*N. gonorrhoeae* can evade the autophagy pathways of host cells during later stages of invasion, which allows a small population of the bacteria to thrive for a prolonged duration and show exocytosis. This may be due to the modulation of autophagy pathway repressor mTORC1 and inhibition of autophagosome maturation and lysosomal fusion (298). Amphiregulin, a protein capable of inhibiting the growth of cancerous cells, is downregulated by *N. gonorrhoeae* during the G1 phase of the cell cycle alongside cyclin degradation (299). It may be noted here that the levels of cyclins were previously measured to identify mechanistic pathways (299). Several other factors such as the ribonuclease L, hereditary prostate cancer 1, and toll-like receptor have also been studied for their role in the development of cancer (300). The double-stranded DNA breaks have also been observed due to the *N. gonorrhoeae* infection, along with the downregulation of p53 (301). In addition, the bacteria produce increased levels of restriction endonucleases during an active infection, ultimately resulting in mutagenesis, which is evidently observed in the form of longer and impaired M-phase of spindle assembly, formation of micronuclei, and lagging of chromosomes (302).

On the contrary, the evidence as mentioned above has been disputed by several studies that could not find a correlation between infection and cancer (303, 304). One of the main challenges associated with the treatment of *N. gonorrhoeae* infection is the development of antimicrobial resistance to standard drugs, including cephalosporins, macrolides, and tetracyclines (305) suggesting the requirement of intensive screening, prevention, or cure for men with gonorrhoea (306).

### *Cutibacterium acnes* (Formerly *Propionibacterium acnes*)

*Cutibacterium acnes* (*C*. *acnes*) is a gram-positive anaerobic bacillus commonly found in the follicles of the skin. In men, a higher prevalence of pro-inflammatory C. acnes has been associated with prostate cancer (26–29). In studies conducted using *in situ* hybridization, clusters of *C. acnes* in 50% of patients with prostate cancer were documented (307). Nonetheless, as a common skin commensal, the presence of *C. acnes* has been regarded as contamination (308). On the contrary, sequence typing of the bacteria indicated them to be urogenital pathogens and not skin commensals (309). Reports suggest that some species of the bacterium have cytotoxic and hemolytic properties (310) and are also noted for the extensive immunomodulatory character (311), revealing factors that interfere with virulence and host tissue (312). The bacterium showcased a wide range of virulence factors, including enzymes such as lipases, proteases, and chemotactic factors for immune cells (313). Reports also indicate the promotion of innate immune cells, including macrophages which release cytokines such as tumor necrosis factor, Il-1, 6, 8, and 12 (27, 314, 315). By upregulating the vascular endothelial growth factor (VEGF), IL-17 release contributes to the activation of malignant cell proliferation and the production of new blood cells (316). Further, the disbalance in IL-17 and regulatory T-cells (Treg) cells in tumors could aggravate oncogenesis by inducing immunosuppression (315, 317).

High antibody titers against *C. acnes* were observed in men with benign prostatic hyperplasia, indicating the existence of infection and inflammation (318). While the infection affects cell proliferation leading to transformation (319), inflammation elicits oncogenesis by enhanced mutagenesis, cell replication, and angiogenesis (320). An increased Th1-type immune response is observed in the site of infection as a result of inflammation (320, 321) which harms the neoplastic process. However, in prostate cancer, proliferation-promoting Th2-type of response was seen (322). Increased nuclear factor-kappa B (NF-κB) activity in tumors, due to increased IkappaB kinase (IB kinase) activity (323), lead to increased expression of several genes known to be crucial for cancer development and progression (324). The levels of serum inflammatory cytokines 1,6, and 8 were found to rise alongside intensified IL-6 secretion (314, 325). IL-6 triggered the JAK signaling pathway, which in turn activates STAT3, whose repeated stimulation was seen to enhance cell proliferation and eventually cancer (326). A prolonged *C. acnes* infection triggered the production of reactive oxygen species (ROS) in cells as well as the influx of immune cells such as macrophages to the infection site along with inhibition of apoptosis (327). Oncogenesis was suggested to be favored by this combination (319).

FOXM1 (Forkhead box M1), a transcription factor linked with cell proliferation and involved in tumorigenesis achieved *via* promoting cell progression into S and M phases, was downregulated during the *C. acnes* infection (328). FOXM1 also facilitated the recombination and repair of double-stranded DNA during breaks, maintenance of stability by control of Aurora B kinase, Cyclin B1, and Centromere protein F. It is speculated that FOXM1 downregulation could lead to mutagenesis, notwithstanding more information is yet to be obtained in this regard (328). Other researchers have proposed the role of androgen levels in cancer development (329), but the signaling pathways remain unclear as of now (330). In murine models, the *in vivo* inoculation of C. acnes showed an inflammatory response eliciting cell damage (331, 332). *In vivo* evidence in the mice model found the prostate cancer generation and following chronic infection of up to 2 months, accompanied by a rise in proliferation and decreased androgen receptor levels (309, 333) and validating the inflammatory theory (332). *C acnes* infection may be treated with antibiotics including (β-lactams, quinolones, clindamycin, or rifampicin, despite increasing evidence of resistance towards these classes. Further, treatment could require surgical intervention to completely eradicate the bacteria (334).

### “*Mycoplasma*” Species

Since the initial hints of an association between Mycoplasmas and oncogenesis, many studies have attempted to understand its oncogenic properties and its direct or indirect role in the onset of cancer or its progression (335–337). There is not much evidence to support current proposed mechanisms for Mycoplasma-induced oncogenesis. Improving diagnosis and tracking of Mycoplasma infections in patients is necessary to improve available data linking infection with pathological and clinical outcomes. Mycoplasma infections can be identified in a timely manner in patients by identifying antibodies induced in the host following infection (338–340).

Yet to be proven, *Mycoplasma pneumoniae* has been suspected as a probable cause of leukaemia since the mid-20^th^ century. The meta-analyses of various cancer studies revealed the possible involvement of Mycoplasma species in oncogenic processes (341). Gliomas, Hodgkin’s, along with non-Hodgkin’s lymphoma, head and neck cancer, as well as cervical cancer have all been linked to Mycoplasma spp (338, 342–344). During the first examination of the etiology and role of venereal diseases in prostate oncogenesis in the 1950s, persistent inflammation and atrophy were suggested as probable processes resulting in the development of prostate cancer (345–348). Mycoplasmas are commonly present in the male urogenital tract, with the most prevalent species being *Mycoplasma hominis* and *Mycoplasma genitalium* (349–351). Current research has looked at the function of mycoplasmas in prostate cancer development. Due to the chronic infections with mycoplasma species in oncogenic cases, their involvement in oncogenesis has been strongly suggested (34–37).

Although altered inflammatory pathways, along with disruption of cell division and DNA repair, have been viewed as possible causes for cancer initiation, the exact mechanisms for cancer formation by mycoplasmas remain unclear (352, 353). Mycoplasmas cause long-term infection and develop immune escape mechanisms by modifying the inflammatory response (353). Infections with mycoplasmas lead to chronicity by a range of strategies that undermine the immune response, including degradation of immune effector molecules, cell invasion, molecular mimicry, antigen variation, and biofilm development, besides inflammatory regulation (354). As a means of immune evasion, the invasion of the host cell may result in the production of proteins that modulate critical cellular processes such as apoptosis and DNA repair. sAs a result of these modifications, the likelihood of aberrant cell development and oncogenicity increases (355). Some Mycoplasmas (notably *M. fermentans*, *M. penetrans*, and *M. hyorhinis*) have been reported to have oncogenic potential due to their ability to cause phenotypic changes in the cells in addition to inducing DNA aberrations (356). Long-term infections with Mycoplasmas are also connected to the instability of chromosomes and neoplastic modifications such as decreased cell adherence, spindle shape, and multilayer growth in cell cultures (357). Although a direct link between Mycoplasma infection and cancer formation remains still explored, the epidemiologic and observational data strongly imply the greater risk of cancer development with Mycoplasma spp. infections (355).

The NLRP3 inflammasome, a protein complex that controls the production of pro-inflammatory cytokines, including IL-1 and IL-18, is also involved in cancer development and spread (358). Many *in vitro* carcinogenic models to demonstrate the cancer-causing abilities of mycoplasmas, have been developed. Mutagenesis, disruption of the cell cycle checkpoints, apoptosis, and altered cell growth signals have been observed to be caused by mycoplasmas (357, 359). As a result, researchers have hypothesized that chronic mycoplasma infections can cause genetic instability and DNA aberrations as a result of mitogenic and apoptotic effects, eventually leading to tumor formation (357, 359, 360). *M. fermentans* and *M. penetrans* demonstrated their capability of oncogenesis promotion in murine CH3 cells showing mutagenic properties *via* the c-Ras and c-myc genes (352, 361). Such cells have been observed to accumulate mutagens and eventually mutate their DNAs due to altered methylation of DNA (362). Mycoplasma DnaK, a chaperone protein from the HSP-70 family, binds to and inhibits the catalytic activity of poly adenosine diphosphate-ribose polymerase (PARP)-1, a protein involved in the detection and repair of DNA damage. It also binds to USP10, an important p53 regulator, compromising p53 stability and anti-cancer potential. Mycoplasma-associated carcinogenic activity, mediated *via* the suppression of DNA repair and p53, may initiate some cancers, albeit not always in later stages (363). The same has been demonstrated *in vivo* in mouse models *via* lymphomagenesis (364). NF-κB activation for the inhibition of p53 is a proposed mechanism of oncogenesis, as demonstrated in the murine model (357). In the tissues of mammals, a lipoprotein present on the surface membrane, P37, is known to play a role in adhesion (351) *via* association with epidermal growth factor receptor 2 (365, 366). *M. hyorhinis* produces the p37 protein, which can promote cancer cell invasion in a dose-dependent manner and blocked by monoclonal antibodies specific for p37. Because p37 makes prostate cancer more aggressive, the molecular events it causes could be a therapeutic target (365). Mycoplasma infection functions as a p53-suppressing oncogene that collaborates with Ras in cell transformation, implying that mycoplasma’s carcinogenic and mutagenic effects are due to its inhibition of p53 tumor suppressor activity (367), which has been demonstrated in several Mycoplasma strains, ultimately resulting in downregulation of apoptosis of the damaged cells. Similar *in vitro* studies performed on human cell lines showed malignancy in prostrate cells, cervical cells, and bronchial cells (365, 368, 369), while other *in vivo* studies have also concluded the species of Mycoplasma to promote oncogenesis (367).

Investigation of small cell lung cancer and mycoplasma association revealed a considerably high mycoplasma presence in the patients compared to healthy groups, which led the researchers to speculate a multistage oncogenesis pathway directed by the bacteria (370), however, more research in this line is required for elucidating its exact role. It was hypothesized that the association between mycoplasmas and renal cell carcinoma mechanism was a persistent infection in the kidneys which stimulated oxidative stress (371).

Although *in vitro* and *in vivo* studies have suggested the involvement of Mycoplasmas in oncogenesis, more studies are needed to decipher its specific role in oncogenesis, diving into cellular and molecular mechanisms involved in the neoplastic transformation. Any role of mycoplasmas in causing tumors needs to be strengthened with more laboratory studies.

### Prevention of Infection-Associated Cancers

Many integrated approaches may be needfully employed to prevent and control the disease based on different mechanisms linked to the origin of cancers *via* bacterial infections. The primary approach is to prevent the infection and eliminate the root causes of infection in healthy individuals, which may be achieved *via* effective vaccination strategies, preventative antibacterial therapy in endemic regions, and/or prevention of persistent infection (372). Secondary prevention may address patients in the pre-clinical or early stages of cancer and prevent tumor progression (373). For example, certain Asian countries have adapted country-wide screening programs to detect stomach cancer (374, 375). Finally, post-therapy monitoring of the patients for relapses is also an efficient method to ensure the quality of life (376, 377).

## Conclusion

Bacterial etiology for cancer has been suspected for many years, yet not much proof has been obtained. Many organisms have been studied concerning their role in oncogenesis. This review lists the possible cancer-causing bacteria and the associated molecular processes through which oncogenesis may be achieved. While chronic inflammation and toxic bacterial neoplastic metabolites have remained the major concerns, further research into the molecular mechanisms of these infectious agents in the process of the cancer formation is of importance. Additionally, several factors pose a challenge for confirming the role of these bacteria in oncogenesis, including multiple etiology, variable periods between the onset of infection, and diagnosis of cancer.

## Author Contributions

All authors listed have made a substantial, direct, and intellectual contribution to the work, and approved it for publication.

## Funding

This research work was partially supported by Chiang Mai University.

## Conflict of Interest

The authors declare that the research was conducted in the absence of any commercial or financial relationships that could be construed as a potential conflict of interest.

## Publisher’s Note

All claims expressed in this article are solely those of the authors and do not necessarily represent those of their affiliated organizations, or those of the publisher, the editors and the reviewers. Any product that may be evaluated in this article, or claim that may be made by its manufacturer, is not guaranteed or endorsed by the publisher.
